# Impact of prior specifications in ashrinkage-inducing Bayesian model for quantitative trait mapping and genomic prediction

**DOI:** 10.1186/1297-9686-45-24

**Published:** 2013-07-08

**Authors:** Timo Knürr, Esa Läärä, Mikko J Sillanpää

**Affiliations:** 1 Department of Mathematics and Statistics, P.O. Box 68, University of Helsinki, Helsinki, FIN-00014, Finland; 2 Department of Mathematical Sciences/Statistics, P.O. Box 3000, University of Oulu, Oulu, FIN-90014, Finland; 3 Department of Biology and Biocenter Oulu, P.O. Box 3000, University of Oulu, Oulu, FIN-90014, Finland; 4 Department of Agricultural Sciences, P.O. Box 27, University of Helsinki, Helsinki, FIN-00014, Finland

## Abstract

**Background:**

In quantitative trait mapping and genomic prediction, Bayesian variable selection methods have gained popularity in conjunction with the increase in marker data and computational resources. Whereas shrinkage-inducing methods are common tools in genomic prediction, rigorous decision making in mapping studies using such models is not well established and the robustness of posterior results is subject to misspecified assumptions because of weak biological prior evidence.

**Methods:**

Here, we evaluate the impact of prior specifications in a shrinkage-based Bayesian variable selection method which is based on a mixture of uniform priors applied to genetic marker effects that we presented in a previous study. Unlike most other shrinkage approaches, the use of a mixture of uniform priors provides a coherent framework for inference based on Bayes factors. To evaluate the robustness of genetic association under varying prior specifications, Bayes factors are compared as signals of positive marker association, whereas genomic estimated breeding values are considered for genomic selection. The impact of specific prior specifications is reduced by calculation of combined estimates from multiple specifications. A Gibbs sampler is used to perform Markov chain Monte Carlo estimation (MCMC) and a generalized expectation-maximization algorithm as a faster alternative for maximum *a posteriori* point estimation. The performance of the method is evaluated by using two publicly available data examples: the simulated QTLMAS XII data set and a real data set from a population of pigs.

**Results:**

Combined estimates of Bayes factors were very successful in identifying quantitative trait loci, and the ranking of Bayes factors was fairly stable among markers with positive signals of association under varying prior assumptions, but their magnitudes varied considerably. Genomic estimated breeding values using the mixture of uniform priors compared well to other approaches for both data sets and loss of accuracy with the generalized expectation-maximization algorithm was small as compared to that with MCMC.

**Conclusions:**

Since no error-free method to specify priors is available for complex biological phenomena, exploring a wide variety of prior specifications and combining results provides some solution to this problem. For this purpose, the mixture of uniform priors approach is especially suitable, because it comprises a wide and flexible family of distributions and computationally intensive estimation can be carried out in a reasonable amount of time.

## Background

Genetic association studies, quantitative trait loci (QTL) mapping and genomic prediction rely on increasingly dense DNA information such as single nucleotide polymorphisms (SNP). The increasing abundance of marker data amplifies one of the essential statistical problems in such studies: the number of potential explanatory variables represented by single markers is often larger than the number of observations in the sample studied, and some regularization is required to ensure the identifiability of the marker effects. Suitable statistical models can accomplish this regularization by variable (i.e. marker) selection, shrinkage of marker effects towards zero or a combination of these two strategies [1-4].

Many variable selection and shrinkage techniques based on Bayesian modelling and Markov chain Monte Carlo (MCMC) algorithms have been proposed for genetic association studies, QTL mapping and genomic prediction (see [5,6]). They differ in the set-up of the statistical model and in their prior specifications. Probably the most popular alternatives are reversible jump MCMC [7-9], stochastic search variable selection (SSVS) [10,11] and locus-indicator models [12]. To avoid some of the complications in model selection, saturated models have been proposed in which genetic effects from all possible explanatory markers are collected simultaneously into the model and their identifiability is increased by prior assumptions that result in shrinkage of effect sizes towards zero [1,4,13]. Such a shrinkage-inducing method leads to a solution in which large effects tend to occur only at rather few positions along the genome in the posterior distribution.

In a previous study, we presented a new class of shrinkage-inducing priors: a mixture of discrete uniform distributions (MU), and compared it to other methods in the context of QTL detection [14]. Compared to methods commonly used in genomic prediction, the main differences and similarities are the following: MU is a shrinkage-based method like BayesA [1] and Bayesian LASSO [13,15], but it is richer in the variety of tuning-parameters. This may be bad from a tuning point of view, but the hyper-parameter combinations in the prior specification potentially covers a wider spectrum of different scenarios concerning the genetic architecture of the trait, heritability, marker spacing or structure of linkage disequilibrium (LD) in the data. Like BayesB [1] and SSVS [10,11], MU includes a hyper-parameter for the prior probability of no marker association, but unlike BayesB and SSVS, the prior of MU does not include any indicator variables. Therefore, use of such separate indicator variables is avoided in the estimation algorithms of MU, which otherwise could negatively affect the speed and the mixing properties during MCMC simulation or cause multimodality problems in maximum *a posteriori* estimation (see [16]).

Bayesian shrinkage methods are common tools in genomic prediction, but rigorous decision making in the context of QTL detection via such models is not well established [17]. Here, we shall examine in more detail the properties of MU, focusing in particular on how robust the results are in the analysis of the well-studied QTLMAS XII data set with tightly linked markers [18,19]. In addition, we test the prediction ability for genomic selection purposes in a real data set on a population of pigs [20]. As suggested in [14], MU appears to be sensitive to prior parameters. In this study, we resume the issue of prior sensitivity and we extend the analysis. As a potential solution to the prior sensitivity issue, we define a finite set of prior specifications and use ”poor-man’s” model averaging over these by giving equal probability/weight to each prior setting. We compare these consensus estimates to the presumably less robust ones from single prior specifications. MU comprises a wide and flexible family of prior distributions, because it is controlled by three hyper-parameters instead of two or one as in most other shrinkage approaches without indicators in the model. Furthermore, the prior assumptions in MU provide a coherent framework for formal hypothesis testing and calculation of Bayes factors, which is lacking in most other shrinkage-based variable selection methods [17]. As another exception with a coherent framework, a decision rule based on Bayes factors has been proposed for the extended Bayesian LASSO [21].

For MCMC simulation of the posterior distribution, we have implemented a Gibbs sampler, for which we provide the fully conditional distributions in Additional file 1 and the C code as an extension module to the software package R [22] in Additional file 2. As a faster alternative to MCMC estimation, we have constructed a generalized expectation-maximization (GEM) algorithm for maximum *a posteriori* (MAP) point estimation [23], for which we provide the estimation details and the C implementation in Additional file 3.

## Methods

### Data model and Bayesian hierarchical set-up

Consider a population-based sample of *N* individuals with phenotype measurements *Y*_*j*_ (j=1,…,N). Suppose each individual has been scored at *M* markers and the genotype observation of an individual at marker *m* (m=1,…,M) is denoted by *x*_*jm*_. Assuming bi-allelic markers such as SNP (single nucleotide polymorphisms) and only additively acting gene effects, genotype observations are coded as −1,0 and 1 corresponding to the three possible genotypes, say *AA*,*Aa* and *aa*.

The phenotype of individual *j* is modelled by the following regression equation

(1) $$Y_{j}=\alpha +\overset{M}{\underset{m=1}{\sum }}\beta_{m}x_{\text{jm}}+\varepsilon_{j}.$$

Here, *α* is the intercept common to all individuals in the population. Furthermore, each *β*_*m*_ holds the additive effect of marker *m*, and *ε*_*j*_ the error term for the individual. A complete description of the distributional assumption made to specify the likelihood as well as its mathematical formula are included as supporting information [see Additional file 1]. Constant variances are assumed for *α* and {*β*_*m*_} in their respective prior specifications, whereas a common random variance *σ*^2^ is assumed for the error terms. Conditional on *σ*^2^, mutual independence is assumed among the other parameters (*α*, {*β*_*m*_}, {*ε*_*j*_}). If appropriate, the regression can readily be extended to include a polygenic component with kinship-based variance-covariance structure to account for infinitesimal marker effects and/or background QTL.

### Prior specifications for shrinkage-based variable selection

As typical in this type of Bayesian variable selection approaches, restrictive shrinkage priors are assigned to the effect size parameters to regularise the model, to avoid overfitting and to ensure the identifiability of genetic marker effects. In the following, we describe such an approach, which provides a mechanism to shrink spurious effect sizes towards 0. We use a mixture of three distinct uniform distributions (MU), the performance of which has been previously evaluated using two well-documented real data sets and comparing it to two other Bayesian variable selection approaches [14]. Since we used the software package OpenBUGS [24] in our previous study to perform MCMC simulation, our report was restricted to samples with much fewer individuals and markers than in this study. Here, we overcome this drawback by a Gibbs sampler implementation for MCMC simulation of the posterior distribution and a GEM algorithm for fast maximum *a posteriori* point estimation in the low-level C programming language.

Both types of algorithms are based on the fully conditional univariate posterior distributions and single parameters are updated one at a time; whereas the Gibbs sampler iterates over random draws from these distributions, GEM only iterates over the fully conditional expected values before reaching convergence in a - possibly local - maximum of the parameter space. For a detailed discussion on GEM and its affinity with standard EM and related algorithms see [25].

The assumptions of the prior distribution are completely specified in the supporting information [see Additional file 1]. In Additional file 1, we also derive the univariate fully conditional posterior distributions needed for a single-site Gibbs sampler and the fully conditional expected values for GEM. The C codes for both algorithms are provided in the supporting information [see Additional files 2 and 3].

In MU, each effect size, *β*_*m*_, is assigned a prior distribution with probability density function

(2) $$\begin{array}{l} p(\beta_{m})=\;p_{0}\cdot \frac{1}{2b}I_{\left(-b,b\right)}(\beta_{m})\\ \;+\frac{1-p_{0}}{2}\cdot \frac{1}{l-b}\left[I_{\left[-l,-b\right]}(\beta_{m})+I_{\left[b,l\right]}(\beta_{m})\right], \end{array}$$

where *I*_*A*_(*x*) is the indicator function of a set *A*, i.e. its value is 1 if *x*∈*A* and 0 otherwise; furthermore, *p*_0_∈(0,1) is the prior probability that *β*_*m*_ obtains a value close to 0 in the interval (−*b*,*b*), with the border value set to *b*>0, and 1−*p*_0_ is consequently the prior probability that *β*_*m*_ lies further away from 0, either in [−*l*,−*b*] or in [*b*,*l*], with the effect size limit set to *l*>*b*. If the three hyper-parameters *p*_0_, *b* and *l* are appropriately chosen, this density has a narrow peak around zero and is flat on the rest of its support. Thus, this density is a step function, resembling a spike and a slab [26]. The slab is sometimes also referred to as a smear (e.g. [27]).

The mixture of three uniform distributions is specified by allocating a major amount of probability mass, *p*_0_, on a small interval (−*b*,*b*) that covers 0 and the remaining probability mass, 1−*p*_0_, on two intervals that lie symmetrically at either side away from 0. Distributing the probability mass in this way reflects the prior perception that a marker chosen arbitrarily from a large set is unlikely to explain a substantial portion of the phenotypic variation. In other words, most marker effects are expected to be so close to 0 that their contributions can be considered negligible.

Biological expert knowledge and practical considerations should determine the choice of the three hyper-parameters. Considering the contribution to the phenotypic variation of effect sizes lying within the spike (|*β*_*m*_|<*b*) as negligible, yields a criterion to discriminate between associated and non-associated markers. However, other aspects such as sample size and coarseness of measurement affect the choice of *b*, because a small sample size and imprecise data reduce the chances to identify small marker effects. If |*β*_*m*_|≥*b* is used as the criterion for QTL identification, the prior belief concerning the total number of associated markers can be directly expressed via the choice of *p*_0_; the number of markers with |*β*_*m*_|≥*b* has *a priori* a binomial distribution with mean *M*(1−*p*_0_) due to the independence assumed among {*β*_*m*_}. The hyper-parameter *l* restricts the absolute effect size of a marker to a certain upper limit, which is difficult to quantify *a priori*, because the genetic architecture of the trait and specifically the distribution of effect sizes are not known. However, empirical studies indicate that effect sizes of more than a few phenotypic standard deviations seem unlikely (see [28-30]).

In the context of regression models for genomic prediction, a rough guideline has been suggested for choosing hyper-parameters in the prior distribution of genetic effects based on a connection between the prior variance of SNP effects and the expected heritability of the trait (cf. [6]). For MU, the variance of the effect of a single SNP can be easily obtained from Equation (2) and integration yields

$$\text{Var}(\beta_{m})=\frac{1}{3}\left[b^{2}+l(l+b)(1-p_{0})\right].$$

Gianola et al. [31] derived that

$$\text{Var}(\beta_{m})=\frac{V_{A}}{2\overset{M}{\underset{m=1}{\sum }}f_{m}(1-f_{m})}$$

under idealized conditions (Hardy-Weinberg equilibrium, linkage equilibrium between QTLs, and QTL positions coinciding with marker positions). Here, *V*_*A*_ is the additive genetic variance and *f*_*m*_ the allele frequency at marker *m*. Under these conditions, the narrow-sense heritability, i.e. *h*^2^=*V*_*A*_/*V*_*P*_ with *V*_*P*_ being the phenotypic variance, can be expressed as

(3) $$h^{2}=\frac{2\text{Var}(\beta_{m})\sum_{m=1}^{M}f_{m}(1-f_{m})}{V_{P}}.$$

As pointed out by de los Campos et al. [6], if the genotypes at each marker are standardized to have a mean of 0 and a variance of 1 instead of using -1, 0, and 1 as genotype codes, the relationship just mentioned becomes

(4) $$h^{2}=\frac{\text{Var}(\beta_{m})M}{V_{P}}.$$

Note that the values of *h*^2^ are not restricted to the interval (0,1) but merely to (0,*∞*). Here, it is noteworthy that altering the genotype codes via standardization affects the interpretation of the effect size estimates, since *β*_*m*_s do not represent additive genetic effects on the phenotype scale in this case.

### Tools of inference

As in our previous study, we calculated the Bayes factor for the hypothesis that the absolute value of the marker effect exceeds a certain threshold value to assess the strength of the association between the phenotype and a single marker *m*. As in any shrinkage-inducing approach, choosing this threshold is arbitrary or needs to be controlled by permutation of the phenotype [4]. In the case of MU, however, the choice of *b* as the threshold results in a framework which is coherent with the prior assumptions concerning the effect size *β*_*m*_, namely that the contribution of markers with effect sizes in the interval (−*b*,*b*) are negligible. By defining an indicator variable *S*_*m*_=*I*_[*b*,*l*]_(|*β*_*m*_|), the posterior probability of the hypothesis can be expressed as *P*(*S*_*m*_=1|data). To obtain the Bayes factor for the two competing hypotheses *H*_1_: *S*_*m*_=1 against *H*_0_: *S*_*m*_=0, the posterior odds is divided by its prior odds [32,33]:

$${\text{BF}}_{m}=\frac{P(S_{m}=1|\text{data})}{1-P(S_{m}=1|\text{data})}/)\frac{P(S_{m}=1)}{1-P(S_{m}=1)},$$

where the prior probability *P*(*S*_*m*_=1)=1−*p*_0_ is readily available from the prior specification of *β*_*m*_ in MU.

Kass and Raftery [32] have suggested the following categories to classify the strength of evidence provided by twice the natural logarithm of the Bayes factor, 2ln(BF_*m*_), as a slight modification to the categories presented by Jeffreys [34]: evidence in favour of the hypothesis is considered very strong for values >10, strong for values in (6,10], positive for values in (2,6], and not worth more than a bare mention for values in (0,2], respectively.

As mentioned above, the choice of a threshold for the effect size *β*_*m*_ is generally problematic in shrinkage approaches, whereas the prior specification of MU entails a justification for a specific threshold in MU. Unless indicator variables are integrated into the likelihood of the model (e.g. as in [35]), most shrinkage approaches do not provide an unequivocal frame of hypotheses necessary for the Bayes factor. A notable exception is the extended Bayesian LASSO [21], where the prior distributions of locus-specific variances depend on regularizing shrinkage parameters, which can be tested for QTL presence via Bayes factors.

Besides the choice of a threshold for *β*_*m*_, another conceptual problem may arise in shrinkage approaches in which improper priors for the effect sizes are used, such as the model proposed in [36] as a modification of the approach in [4]; although the posterior probability *P*(*S*_*m*_=1|data) and consequently the posterior odds may exist also for improper priors, the prior odds is not available for the complementary hypotheses *b*<|*β*_*m*_| vs. |*β*_*m*_|≤*b*, because the integral over the prior distribution corresponding to the former hypothesis does not exist.

We assessed the sensitivity of single analyses by comparing results under varying prior specifications, and for MCMC additionally under identical prior specifications to detect convergence or mixing problems. In addition, we combined Bayes factor information from different analyses to increase the robustness in detecting association signals.

We also evaluated the predictive abilities of our model by comparison of genomic estimated breeding values (GEBV) either with the true breeding values (TBV), as available in simulated data sets, or with the phenotype measurements directly, as available in real data sets. The GEBV for individual *i* is

$${\text{GEBV}}_{i}=\overset{M}{\underset{m=1}{\sum }}{\hat{\beta }}_{m}x_{\text{im}},$$

where ${\hat{\beta }}_{m}$ is the posterior mean of *β*_*m*_ in the case of MCMC or the MAP point estimate in the case of the GEM algorithm, and (*x*_*im*_) is the vector of genotype codes for the individual. For cross-validation of our results, we employed the faster GEM algorithm. Also here, we compared estimates from single prior specifications with combined estimates from multiple ones. A more detailed description of these procedures is given in the following sections.

### Analysis of the simulated QTLMAS XII data

This simulated data set was originally distributed as a part of the 12th European workshop on QTL mapping and marker assisted selection (QTLMAS XII) held in Uppsala, Sweden, on 15–16 May 2008. Detailed information on the publicly available data [37] has been presented by Crooks et al. [18] and Lund et al. [19].

The simulation of the phenotype involved a total of 50 bi-allelic QTLs with additive effects. Crooks et al. [18] classified 15 of these as major QTL (denoted by M1-M15), because they yield *P*-values of less than 0.05 after Bonferroni correction in a multiple linear regression including all genotypes of true QTLs. The whole data set available for QTL detection consists of 4665 individuals from a pedigree of consecutive generations. We excluded the 165 individuals of the first generation from our analysis, because they do not form full-sib families of size 10 like the 4500 individuals in the subsequent generations. The founders of each generation were 15 males and 150 females. In the first generation, all individuals were used as parents, whereas in the second and third generation, they were randomly sampled. Each male parent was mated to 10 females, each producing 10 full-sib offspring. Thus, the pedigree actually has a full-sib and half-sib structure. However, we did not take into account the familial resemblance between half-sibs or between parents and offspring from consecutive generations in our statistical model.

For simplicity, we merely considered polygenic family effects (*u*_*k*_) for full-sib families and extended the regression in Equation (1) to

$$Y_{\text{kj}}=\alpha +\overset{M}{\underset{m=1}{\sum }}\beta_{m}x_{\text{kjm}}+u_{k}+\varepsilon_{\text{kj}},$$

for individual *j* ($\;j=1,\ldots ,N_{k}$) from family *k* (k=1,…,K). The polygenic terms *u*_*k*_ were assumed conditionally independent random effects with a mean of 0 and a common random variance $\sigma_{u}^{2}$.

Our results are based on *N*=4500 individuals in *K*=450 full-sib families, each of size *N*_*k*_=10. The marker data consists of 6000 completely genotyped SNP equidistantly spaced by 0.1 cM spanning six chromosomes with 1000 markers each. We removed the 106 markers with minor allele frequency of less than 0.01, yielding *M*=5894 markers for analysis of the complete genome.

#### Association mapping

We ran MCMC simulations for four different sets of prior specifications (see details in Table 1). Our first goal was to evaluate the power of MU to detect QTL and the false positive error rate in this data set with tightly-linked markers and to compare the findings with the results from the six association studies reported in [18]. Secondly, we aimed at assessing the robustness of our results in several MCMC runs under identical and varying prior specifications. For each set of prior specifications, we started two MCMC chains from different starting values. Thus, the results are based on a total of eight chains (marked by A-H). In each run, we simulated 220 000 Gibbs iterations, of which the first 20 000 were discarded as burn-in. This burn-in size was determined based on informal convergence checks. We applied thinning to save disk space and only stored every 20th iteration. Thus, each of the eight runs yielded 10 000 MCMC samples for the analysis of the joint posterior distribution. The MCMC simulation of a single chain took 6 - 6.5 hours on a computer with a 3 GHz dual core processor and a physical memory of 2 GB. All simulations shared the following prior specifications: the upper limit of the effect size parameters *β*_*m*_ was set to *l*=sd(*Y*)=2.10, the prior variance of the common intercept *α* to *c*=10^6^, and the shape and rate parameters (*s*_*u*_,*r*_*u*_,*s*,*r*) were all set to 0.01 in the inverse-gamma distributions used as priors of the variance components $\sigma_{u}^{2}$ and *σ*^2^ [see Additional file 1 for the parametrisation of the inverse-gamma distribution]. For an inverse-gamma distribution with shape parameter *s* and rate parameter *r*, its mean has the value $\frac{r}{s-1}$, if *s*>1, and its variance has the value $\frac{r^{2}}{{\left(s-1\right)}^{2}(s-2)}$, if *s*>2. Thus, the mean and variance do not exist for our choice of shape and rate parameters because of a heavy right tail. However, the mode exists, with a value of $\frac{r}{s+1}=\frac{1}{101}$. With both *r* and *s* decreasing towards 0, the inverse-gamma distribution approaches the noninformative scale-invariant, but improper prior with density ∝1/*σ*^2^.

**Table 1:** Comparison of the prior specifications in the eight MCMC chains A-H used to analyse the QTLMAS XII data, posterior estimates of model parameters and summary statistics

	**Prior specification**			**Posterior mean (sd) of**				
**Chain**	***p***_***0***_	***b***^***(a)***^	***N***_***Q***_	***α***	$10^{2}\sigma_{u}^{2}$	***σ***^***2***^	***hM2***^***(b)***^	***N***_***Q***_
A	0.99	0.01	58.9	2.0 (0.6)	1.7 (1.2)	3.0 (0.1)	0.32 (0.02)	23.0 (2.5)
B	0.99	0.01	58.9	2.6 (0.7)	1.7 (1.2)	3.0 (0.1)	0.32 (0.02)	22.9 (2.6)
C	0.99	0.001	58.9	2.3 (0.5)	3.0 (1.9)	3.0 (0.1)	0.30 (0.02)	31.5 (2.5)
D	0.99	0.001	58.9	2.6 (0.5)	3.0 (1.9)	3.0 (0.1)	0.29 (0.02)	31.1 (2.5)
E	0.999	0.01	5.9	2.1 (0.5)	1.9 (1.3)	3.0 (0.1)	0.31 (0.02)	15.3 (1.3)
F	0.999	0.01	5.9	2.8 (0.5)	2.1 (1.4)	3.0 (0.1)	0.31 (0.02)	14.3 (1.3)
G	0.999	0.001	5.9	1.9 (0.4)	3.9 (2.3)	3.1 (0.1)	0.28 (0.02)	21.5 (1.4)
H	0.999	0.001	5.9	2.0 (0.7)	3.7 (2.2)	3.1 (0.1)	0.28 (0.02)	22.6 (1.8)

^(*a*)^ given in units of phenotypic standard deviations (sd(*Y*)=2.10).

^(*b*)^ The true overall heritability of the trait is 0.30 [19].

Hyper-parameter *p*_0_ defines the prior probability that the effect size lies in the interval of the spike, (−*b*,*b*). *N*_*Q*_ is a summary statistic for the number of QTL (see text for details), *α* the common intercept in the regression, $\sigma_{u}^{2}$ the variance component of the polygenic terms, *σ*^2^ the residual variance, and $h_{\text{M}}^{2}$ the part of the heritability due to marker effects.

#### Genomic prediction

In addition to the four generations used for QTL detection, the QTLMAS XII data spans over three more generations, providing a validation set for genomic prediction models. Each of these generations holds 400 individuals with complete genotype information and TBV.

To assess the predictive abilities of our model, we first calculated GEBV for the validation individuals, using the posterior means of the effect sizes, *β*_*m*_, from the MCMC chains. For simplicity, the estimated family effects, *u*_*k*_, reflecting pedigree information within the training generations, were not taken into account, because the polygenic effect was negligible in our analysis (see Results section), as well as in a previous study [25]. Furthermore, the family effects were estimated for full-sib families within the training generations and could thus not be applied to the individuals in the validation generations.

We evaluated these GEBV for single prior specifications and their averages across the four prior specifications considered. As in [19], we assessed the predictive ability of the GEBV in the validation individuals by three measures: the accuracy was estimated as the Pearson correlation between GEBV and TBV; in addition, the Spearman rank correlation was calculated between GEBV and TBV for the 10% of the individuals with the largest TBV; finally, the bias of GEBV was estimated as the coefficient of regression of TBV on GEBV.

We also obtained GEBV from the GEM algorithm and assessed their predictive ability as just described. Again for simplicity, we excluded the family effects, *u*_*k*_, from the model. Instead of using the original phenotype and genotype information, we standardized the phenotype and the genotype codes at each SNP to have a sample mean of 0 and a variance of 1 in the training set. The GEBV were then estimated as above and translated back to the original scale. The GEM algorithm for one prior specification required 3 to 14 seconds and 19 to 125 iterations to converge on the same computer as mentioned above (with a 3 GHz processor and 2 GB memory). Convergence was declared when the sum of deviations between current and updated parameter values was smaller than (*M*+2)×10^−7^, where *M*+2=5896 is the number of parameters in the model.

As TBV are only available in simulated data sets, we also applied a cross-validation (CV) approach as a method to assess predictive ability of the model in real data sets. Here, we used only the 4500 individuals in the three training generations. Specifically, we used two different 10-fold CV strategies: (I) we randomized the data into 10 distinct validation sets, each holding 45 full-sib families, i.e. all members of a family belonged to the same validation set; (II) each of the 10 full-sibs of a family was randomly assigned to a different validation set. To predict GEBV for the individuals of a single validation set, the other nine sets were combined to form the training set. We divided the correlation between GEBV and phenotype by the square root of heritability $h=\sqrt{0.30}$[19] to convert it to an estimate of the accuracy of the GEBV. The bias of GEBV was estimated as the coefficient of regressing phenotype on GEBV.

### Analysis of the real data

To test the predictive ability of our method in real data, we analysed a pig data set made available by Pig Improvement Company (a Genus company) to the scientific community [20]. Here, we used one of the five phenotypes provided (T5), which was recorded for 3184 genotyped individuals and for which a heritability of 0.62 was reported in [20]. Before analysis, the trait was standardized to have a sample mean of 0 and a standard deviation of 1.

A total of 52 843 SNP were contained in the genotype data made public. The original genotype codes were 0, 1, and 2 for the three SNP genotypes, respectively, and for missing genotypes (< 1%), a non-integer between 0 and 2 had been imputed (see [20] for details). For our analysis, genotype codes were standardized to have a mean of 0 and a standard deviation of 1 at each SNP. Here, we used four subsets of these SNP: (i) a random set of 10 000 SNP from the entire SNP data; (ii) a random pick of 1000 SNP from the set in (i); (iii) a subset of 10 000 SNP, each with a minor allele frequency > 0.05 and filtered from the entire SNP data by sure independence screening (SIS) of the marginal correlations between the phenotype and SNP [38]; (iv) a subset of 1000 SNP, also each with a minor allele frequency > 0.05 and filtered from the entire SNP data by SIS; this was a subset of the set in (iii). Note that the set of 10 000 SNP filtered by SIS is identical to the one used in [25]. We report results including prediction accuracies for all four sets of SNP (i)-(iv).

As the results obtained from other Bayesian approaches were shown to be nearly unaffected by the inclusion of pedigree information in this data set [25], we chose not to include a polygenic component in this part of the analysis. For parameter estimation, we applied the GEM algorithm and considered numerous combinations of the hyper-parameters *p*_0_ and *b*, which ranged from 0.9 to 0.9999 and from 0.0001 to 0.036, respectively. The hyper-parameter *l* was kept constant at 2.

The accuracy of GEBV was estimated by their correlation with phenotypic values divided by the square root of the reported heritability, i.e. $\sqrt{0.62}$. The breeding value of an individual was predicted via 10-fold cross-validation, in which each individual was randomly assigned to one of 10 subsets. Each of these subsets was used once as the validation set, with the other nine subsets forming the training set. By using the same subsets as in [25], our results are directly comparable to the ones obtained in that study. We also obtained an estimate for the bias of GEBV as the coefficient of regression of the phenotype on GEBV. It is important to note that, similar to [25], the pre-selection by SIS was done using *all* individuals, i.e. it was influenced not only by training but also by validation individuals, and may have caused the subsequent cross-validation procedure to over-estimate the accuracies.

## Results

### QTL detection in the QTLMAS XII data

#### Comparison of common model parameters

We begin with an overview of the posterior estimation for the model parameters, with the exception of marker-specific parameters and compare results obtained from the eight MCMC chains A-H. Table 1 shows the varying prior specifications of the MCMC chains and posterior results for model parameters and summary statistics. The values for the border parameter, *b*, are given in units of phenotypic standard deviations (sd(*Y*)=2.10). We defined a summary statistic for the number of QTL based on the marker-specific indicator variables by $N_{Q}=\overset{M}{\underset{m=1}{\sum }}S_{m}$, and for the heritability due to marker effects by $h_{M}^{2}=1-(\sigma^{2}+2\sigma_{u}^{2})/\text{var}(Y)$, where the sample variance var(*Y*) was used as an approximation of the phenotypic variance, ignoring the relatedness between the individuals studied. Here, the variance component $\sigma_{u}^{2}$ of the polygenic effects was multiplied by a factor 2, because the coefficient of the additive genetic covariance between full sibs is 1/2 (see e.g. chapter 7 in [39]).

For the common intercept, somewhat higher deviations of the posterior results were observed between chains with identical prior specifications when the border value of the effect sizes was set to *b*=0.01 (chains A vs. B and E vs. F) than when it was set to *b*=0.001 (chains C vs. D and G vs. H). Thus, at least for these parameters, the prior specification *b*=0.001 yielded more robust results.

All chains produced virtually identical estimates for the residual variance *σ*^2^. The point estimates for the between-family variance $\sigma_{u}^{2}$ were of about two orders of magnitude smaller than *σ*^2^. This indicates that the polygenic effects, *u*_*k*_, absorbed rather little phenotypic variation in the simultaneous analysis of all chromosomes, which is consistent with the results reported by Lund et al. [19]. Since the genetic variation in the data was explained almost completely by the marker effects, little information would be lost if the polygenic terms were excluded from the model. In the analysis of only one chromosome, the polygenic terms played a more influential role (results not shown), since they can absorb genetic effects from the rest of the genome (cf. [40]). Although the estimates for $\sigma_{u}^{2}$ were small when analysing the complete genome, we observe differences between prior specifications: more phenotypic variation was explained by the polygenic effects when *b*=0.001, i.e. in chains C, D, G and H, since $\sigma_{u}^{2}$ obtained larger posterior means in these chains than in the others. This also explains the slightly higher estimates of $h_{M}^{2}$ for *b*=0.01. Here, we should note that the true heritability of the trait for the full pedigree data is 0.30 [19], which closely coincides with our estimates, which ranged from 0.27 to 0.32.

Estimates of the summary statistic *N*_*Q*_ for the number of QTL were, as expected, higher for the chains with *p*_0_=0.99, i.e. with a smaller prior probability of marker exclusion. Here we note that the prior mean of *N*_Q_ is *M*·(1−*p*_0_). Thus for *p*_0_=0.99, the posterior mean values between 24 and 33 were lower than the prior mean of 60. In contrast, the prior mean of *N*_Q_ was 6 for *p*_0_=0.999, but the posterior means were larger with values ranging from 14 to 22. In this sense, the intuition that the prior specifications with *p*_0_=0.999 are more conservative is confirmed. We also observed that the chains with *b*=0.01 produced lower posterior means of *N*_*Q*_ for fixed *p*_0_. This result is intuitive also, since marker indicators are expected to reach the value 1 more easily, when the interval (−*b*,*b*) is shortened.

#### Marker-specific results

Two of our main goals were (1) to assess how well MU identifies true QTL in this data set and (2) to evaluate the risk of false positive QTL detection when applying the Bayes factor as the measure of the evidence in favour of marker association. In Table 2, the 20 markers with the strongest signals in our analysis are listed. Here, we used the following criterion to rank the strengths of association from all *M*=5894 markers: for each marker, we calculated the Bayes factor for the hypothesis *S*_*m*_=1 (see above, *Tools of inference*) in each of the eight MCMC chains A-H. Next, we ranked the Bayes factors within each chain and calculated a marker-specific mean rank across chains as a measure to summarize information from the eight chains. This was done to increase the robustness in assessing the strength of evidence by making the results less dependent on the specific choices of the hyper-parameters in single MCMC chains.

**Table 2:** The 20 markers with the strongest signals of association across chains in the analysis of the QTLMAS XII data

**Marker**			**Closest true** **major QTL**^(***a***)^			**2ln(BF*m***)			**|*β***_***Q***_**|**	***E***_**post**_**(*β***_***m***_**)**			***%*PVE*Q***	***E*post( *% *PVE *m *)**		
**Chr**	**Pos**	**MAF**	**MAF**	**Name**	**Dist**	**avg**	**min**	**max**		**avg**	**min**	**max**		**avg**	**min**	**max**
1	19.5	0.28	0.28	M1	0.50	30	28	32	0.62	0.60	0.59	0.61	3.5	3.4	3.2	3.5
1	40.1	0.09	0.07	M2	-0.10	15	9	21	0.56	-0.35	-0.46	-0.17	0.9	0.6	0.3	0.8
1	77.7	0.28	0.29	M3	-0.47	28	22	32	0.37	0.43	0.41	0.46	1.3	1.7	1.6	1.9
2	26.9	0.44	0.44	M4	0.51	12	10	15	0.35	0.22	0.15	0.28	1.4	0.9	0.5	1.3
2	28.2	0.24	0.44	M4	-0.79	12	7	18	0.35	0.20	0.09	0.33	1.4	0.6	0.3	1.2
2	48.2	0.38	0.40	M6	0.42	25	14	32	0.37	-0.41	-0.45	-0.36	1.5	1.8	1.5	2.2
2	72.9	0.11	0.18	M7	2.01	11	8	15	0.50	0.15	0.04	0.24	1.6	0.2	0.1	0.4
3	13.2	0.33	0.40	M8	1.71	11	7	18	0.30	0.14	0.03	0.28	1.0	0.4	0.1	0.9
3	14.8	0.39	0.40	M8	0.11	8	6	12	0.30	-0.04	-0.08	-0.01	1.0	0.1	0.0	0.3
3	54.1	0.27	0.07	M9	5.90	11	6	16	0.68	0.12	0.01	0.21	1.3	0.3	0.0	0.5
3	60.1	0.16	0.07	M9	-0.10	24	20	29	0.68	-0.39	-0.41	-0.37	1.3	1.0	0.9	1.1
4	75.7	0.05	0.41	M12	0.36	26	12	32	0.58	-0.72	-0.78	-0.58	3.7	1.1	0.9	1.3
4	76.4	0.46	0.41	M12	-0.34	30	28	32	0.58	0.64	0.61	0.67	3.7	4.6	4.2	5.1
4	85.9	0.18	0.41	M12	-9.84	10	9	12	0.58	0.12	0.02	0.19	3.7	0.3	0.0	0.5
4	96.4	0.27	0.19	M13	0.09	13	11	14	0.29	-0.19	-0.27	-0.06	0.6	0.5	0.1	0.8
4	96.6	0.18	0.19	M13	-0.11	9	4	16	0.29	0.11	0.02	0.28	0.6	0.3	0.0	0.7
4	98.3	0.23	0.19	M13	-1.81	9	5	13	0.29	-0.08	-0.20	-0.02	0.6	0.2	0.0	0.4
5	4.2	0.19	0.21	M14	0.95	8	6	11	0.18	-0.06	-0.15	-0.01	0.2	0.1	0.0	0.3
5	93.4	0.36	0.26	M15	0.10	30	28	32	0.75	-0.71	-0.73	-0.68	5.0	5.3	4.9	5.6
5	94.5	0.09	0.26	M15	-1.00	22	15	32	0.75	-0.50	-0.53	-0.48	5.0	1.0	0.9	1.1

^(a)^The three true major QTLs missing are:

M5 on chr. 2 at pos. 30.00 (MAF =0.21, |*β*_*Q*_|=0.33, %PVE_*Q*_ =0.8),

M10 on chr. 4 at pos. 3.21 (MAF =0.39, |*β*_*Q*_|=0.61, %PVE_*Q*_ =4.0),

M11 on chr. 4 at pos. 36.93 (MAF =0.24, |*β*_*Q*_|=0.34, %PVE_*Q*_ =1.0).

Chr = chromosome, Pos = position in cM from the start of the chromosome, MAF = minor allele frequency, Dist = directed distance in cM of a marker to the closest true major QTL, 2 ln(BF_*m*_) = posterior mean of the 2 ×log-transformed Bayes factor in favor of marker association, |*β*_*Q*_| and *E*_post_(*β*_*m*_) = true absolute value and signed posterior mean of the additive effect size, respectively, % PVE_*Q*_ and *E*_post_(%PVE_*m*_) = true value and posterior mean of the percentage of variance explained, respectively.True values are taken from Table one in [18].

For each of these 20 markers, their position in the genome, minor allele frequency and distance to the closest true major QTL are given in Table 2 (cf. Table one of [18]). The minor allele frequencies of the true QTL were added as a reference. The table also provides the posterior means of 2 ln(BF_*m*_)*averaged across chains* as a consensus measure of evidence, the minimal and maximal means across the chains, and the absolute values of the effect sizes (|*β*_*Q*_|) for the true major QTL as reported in [18]. Here we should note that, in the case of a single value of an effect size, it is sufficient to report only the absolute value, since the sign of the value will depend on the genotype coding of the data set. Of course, our estimates also depend on the genotype coding. Nevertheless, we report the signed posterior means of the effect sizes, *E*_post_(*β*_*m*_), from our analysis, because the minima and maxima from the eight MCMC chains could have opposite signs – although this did not happen for the 20 markers reported. Finally, the posterior means of the percentage of phenotypic variance explained are given in Table 2. They were calculated by $E_{\text{post}}\left(\%\text{PVE}\right)=2{\text{MAF}}_{m}(1-{\text{MAF}}_{m})E_{\text{post}}\left(\beta_{m}^{2}\right)/\text{var}(Y)$. Here, MAF_*m*_ is the minor allele frequency of marker *m*, $E_{\text{post}}\left(\beta_{m}^{2}\right)$ the posterior mean of $\beta_{m}^{2}$, and var(*Y*) is as defined above. Note that *E*_post_(*%*PVE) are estimates for single markers and simply summing them up does not yield an estimate for the entire proportion of variance explained by markers, as covariances due to LD between markers are missed in this sum. However, the proportion of variance accounted for by the regression on markers is captured in our estimates $h_{M}^{2}$ (see Table 1).

### Identification of true QTL by Bayes factors and false positives

Twelve of the 15 major true QTL were located within 5 cM from the markers reported in Table 2. In the comparative study of six association analyses, Crooks et al. [18] considered a QTL to be identified correctly if a positive signal was reported within 5 cM from the QTL. The most successful study by Ledur et al. [41] detected 11 true major QTL (see Table four in [18]). No study compared in [18] identified the true major QTL M7, whereas we found a marker with a signal of association within 2.01 cM of that QTL. The only study identifying M9 was Ledur et al. [41], who found an association with exactly the same marker as we did, namely at 60.1 cM on chromosome 3. Another QTL, M14 at 5.15 cM, was identified by only one study: Bink and van Eeuwijk [42] detected a signal at 2.0 cM, but the marker we identified at 4.2 cM is somewhat closer to this QTL.

Three true major QTL, namely M5, M10 and M11, are absent from Table 2. M5 is very close to M4, at 2.59 cM from M4 at position 30.00 cM on chromosome 2. Each of the six analyses compared in [18] identified either M4 or M5 only. M10 at position 3.2 cM on chromosome 4 was identified by all six studies and explained 4% of the phenotypic variance. It is therefore quite intriguing that our results regarding M10 contrast so markedly. M11 was identified only by Cleveland and Deeb [43].

In the list of the 20 markers with the strongest signals in our analysis, two markers were more than 5 cM from a major true QTL and would have been considered false positives in [18]: one of them, at position 54.1 cM on chromosome 3, was 5.9 cM from M9 (at 60.00 cM), and the other, at 85.9 cM on chromosome 4, was located about midway between M12 (at 76.06 cM) and M13 (at 96.49 cM).

Up to now, we have considered an arbitrary number, namely 20, of markers showing the strongest signals of association across different MCMC chains. In many empirical studies, a decision making tool is used to classify markers into two groups: markers with ”significant” and ”non-significant” QTL signals. For this purpose, one can apply a threshold of, say, 10 to the average of 2 ln(BF_*m*_) across the chains when multiple chains are considered. Sixteen of the markers shown in Table 2 fulfil this criterion and four do not. In addition to the three true major QTL mentioned above (M5, M10, M11), M14 would also remain unidentified if this criterion was used. Moreover, the markers at 54.1 cM on chromosome 3 and at 85.9 cM on chromosome 4 would still be false positives, with both Bayes factors exceeding the threshold.

Three of the six analyses compared in [18] produced no false positive signals. To achieve this level of type I error, the threshold has to be set to 12 in our analysis. This would result in missing two additional QTL (M7 and M8) and the total number of detected QTL would decrease to nine. One study (with no false positives) detected more QTL, namely that of Ledur et al. [41], with 11 QTL. However, this study also exploited haplotype information.

### Robustness of marker-specific results

As shown in Table 2, the Bayes factors varied rather little across chains for some markers and a lot for others: e.g. the minimal and maximal 2ln-transformed Bayes factors were 28 and 32, respectively, for the marker at 19.5 cM on chromosome 1, but were 4 and 16 for the marker at 96.6 cM on chromosome 4. Thus, the latter marker showed very strong evidence in one chain but ”only” positive evidence in another one, according to the classification by Kass and Raftery [32].

To quantify the robustness among the eight MCMC chains, we calculated pairwise Spearman’s rank correlation coefficients *ρ* between the chains for the 20 Bayes factors reported in Table 2 (see the upper right triangle in Table 3). When comparing chains with identical prior specifications, the strongest pairwise agreement was observed between chains A and B (*p*_0_=0.99 and *b*=0.01), with a correlation of 0.99, and the weakest agreement between chains C and D (*p*_0_=0.999 and *b*=0.001), with a correlation equal to 0.84. For chains with different prior specifications, the correlation coefficient obtained its lowest value, 0.67, between chains C and E, which differ in both *p*_0_ and *b*.

**Table 3:** Comparison of Bayes factors in the eight MCMC chains to analyse the QTLMAS XII data

	**MCMC chain I**							
**II**	**A**	**B**	**C**	**D**	**E**	**F**	**G**	**H**
A	.	0.99	0.74	0.89	0.89	0.87	0.79	0.78
B	1.00	.	0.72	0.88	0.90	0.88	0.79	0.76
C	1.00	1.00	.	0.84	0.67	0.70	0.70	0.78
D	0.86	0.86	0.93	.	0.82	0.85	0.83	0.78
E	0.84	0.83	0.95	1.02	.	0.96	0.88	0.82
F	0.87	0.86	0.98	1.04	1.03	.	0.89	0.82
G	0.72	0.71	0.77	0.83	0.85	0.84	.	0.90
H	0.68	0.68	0.73	0.80	0.81	0.81	0.98	.

Pairwise comparison of the eight MCMC chains A-H by Spearman’s rank correlation coefficient *ρ* (upper right triangle) and mean ratio of the 2 × log-transformed Bayes factors in chain I vs. chain II (lower left triangle) for the 20 markers with the strongest signals of association across chains in the analysis of all chromosomes by MU.

We also report the ratios of the 2 × log-transformed Bayes factors averaged across the 20 markers for pairs of chains in the lower left triangle of Table 3. These mean ratios give an indication of the differences in magnitude of the Bayes factors between the chains. On average, chains A, B and C yielded the largest Bayes factors of about the same magnitude. The largest differences in Bayes factors were observed between chains A and H and between chains B and H, both having mean ratios equal to 0.68.

### Genomic prediction in the QTLMAS XII data

The comparison of GEBV and TBV in the three validation generations showed accuracies between 0.79 and 0.90 for MCMC-based and GEM-based estimation under the four prior specifications considered (see Table 4). The Bayesian genomic prediction approaches compared in [19] achieved accuracies ranging from 0.84 to 0.92 and the methods compared in [25] from 0.70 to 0.90. The best results reported by [44] and [45] (0.90 and 0.88, respectively) fall within this range. The accuracy of GEBV obtained by frequentist G-BLUP estimation has previously been reported at 0.75 and of GEBV obtained from Bayesian G-BLUP at 0.76 [25]. The rank correlation between GEBV and TBV for the 10% of individuals with the highest TBV ranged from 0.42 to 0.57, whereas [19] reported corresponding values between 0.46 and 0.56. Estimates of bias ranged from 0.84 to 0.98 in our analyses and from 0.85 to 0.98 in [19]. The accuracies from GEM were somewhat lower than from MCMC for single prior specifications, but averaging the GEBV across prior specifications, i.e. the combined estimates, yielded similar accuracy estimates, 0.89 for MCMC and 0.88 for GEM. Notably, the combined estimate for GEM was higher than any of the estimates from single prior specifications. Likewise, the rank correlations ranged from only 0.42 to 0.51 for GEM under single prior specifications, while the combined estimate was 0.53. The corresponding value for MCMC was again somewhat higher (0.56). In contrast, GEM yielded a slightly better value (0.98) for the combined estimate of bias, i.e. the regression coefficient was closer to 1, than the estimate of bias from MCMC (0.94).

**Table 4:** Comparison of genomic estimated breeding values (GEBV) and true breeding values (TBV) and predictive ability via cross-validation under varying prior specifications for the QTLMAS XII data

**Prior specification**		**Accuracy*a***		**Rank correlation*b***		**Bias*c***		**GEM Cross-validation*d***			
***p***_***0***_	***b***	**MCMC**	**GEM**	**MCMC**	**GEM**	**MCMC**	**GEM**	***r***_***I***_	***r***_***II***_	***b***_***I***_	***b***_***II***_
0.99	0.01	0.87	0.80	0.56	0.42	0.91	0.87	0.92	0.93	0.97	0.98
0.99	0.001	0.88	0.87	0.49	0.51	0.88	0.86	0.94	0.94	0.97	0.97
0.999	0.01	0.88	0.79	0.57	0.41	0.94	0.88	0.91	0.93	0.99	1.00
0.999	0.001	0.90	0.84	0.54	0.51	0.92	0.84	0.92	0.91	0.96	0.95
Combined ^*e*^		0.89	0.88	0.56	0.53	0.94	0.98	0.96	0.96	1.05	1.04

^a^Pearson correlation between GEBV and TBV, for MCMC chains and generalized EM algorithm (GEM);

^b^Spearman rank correlation between GEBV and TBV for the 10% of the individuals with highest TBV;

^c^Slope Coefficient from regressing TBV on GEBV;

^d^Accuracy (*r*: Pearson correlation of GEBV and phenotype, divided by the square root of heritability) and bias (*b*: slope coefficient from regressing the phenotype on GEBV) as estimated from two 10-fold cross-validation approaches: (I) entire families assigned together to folds and (II) individuals from the same family assigned to different folds;

^e^Combined estimates obtained by averaging GEBVs across prior specifications.

The accuracies obtained by cross-validation within the first three generations via GEM were higher (*r*_I_ and *r*_II_ from 0.91 to 0.96) than those reported above for the three validation generations. This result supports the expectation that accuracy of GEBV declines for genetically more distant individuals. We did not observe clear differences in the results between the two cross-validation approaches, although keeping the individuals from an entire family together in the same validation set (approach I) increases the genetic distance between training and prediction sets more than assigning individuals from the same family to different validation sets (approach II).

Also for cross-validation, averaging GEBV across prior specifications improved accuracy, when compared to single prior specifications. However, averaging increased estimates of bias (*b*_I_ and *b*_II_), from values below 1 for single prior specifications to values above 1 for the combined estimates.

Finally, we used Equations (3) and (4) to calculate values of *a priori* heritability under the four different prior specifications. As mentioned above, Equations (3) and (4) do not restrict *a priori* heritability to the range from 0 to 1. For the non-standardized genotype codes used in MCMC estimation, the four prior specifications as ordered in Table 4 correspond to *h*^2^-values of 7.9, 7.7, 0.86 and 0.78, respectively. For the standardized genotype codes used in GEM estimation, the corresponding *h*^2^-values are 20.0, 19.7, 2.2 and 2.0, respectively. Thus, impossible values for heritability, i.e. with values above 1, were implicitly assumed in most of the prior specifications. However, reasonable estimates of accuracy were obtained in all cases and the *a priori* heritabilities varied far more in magnitude than estimates of accuracy.

### Genomic prediction in the real data

For the tested combinations of *p*_0_ from 0.9 to 0.9999 and *b* from 0.0001 to 0.036, the best accuracy, with a value of 0.631, was obtained with *p*_0_=0.9999 and *b*=0.008 for the set of 10 000 random SNP (RAND10K). For this combination of *p*_0_ and *b*, the accuracy for the 10 000 SNP filtered by SIS (SIS10K) was only slightly lower, with a value of 0.623. The highest value reported in [25] was 0.63 for two Bayesian model variants with hierarchical Laplace shrinkage priors. In the same study, the accuracy for Bayesian G-BLUP was reported at 0.63. The prior expectation about heritability (cf. Equation (4)) under idealized conditions corresponds to a value of 1.6 for this specific prior specification and 10 000 SNP. It is noteworthy that values of *b*<0.008 yielded lower accuracies, despite the fact that they corresponded to more realistic, i.e. lower, values of heritability, which was 0.62 for this trait.

**Figure 1:**
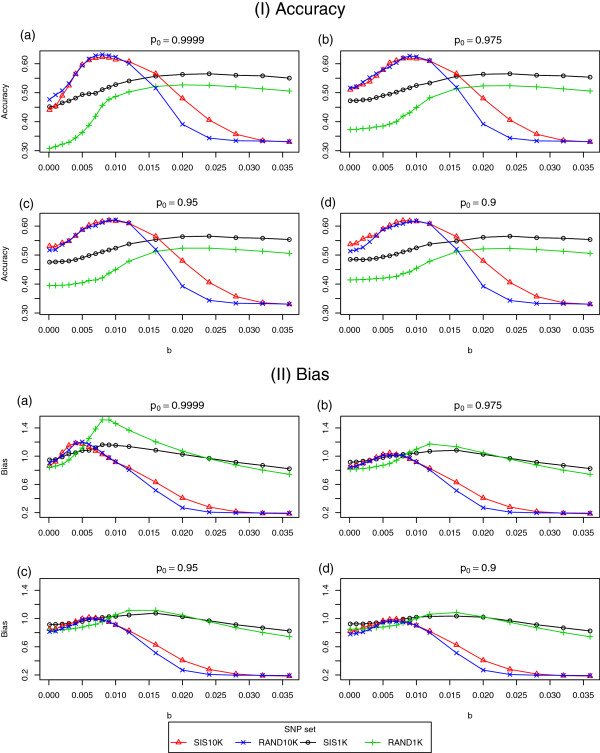
**Accuracy (panel I) and bias (panel II) estimates under varying specifications of the hyper-parameters*p***_**0**_** (subpanels a-d for both panel I and II) and*b* for the four SNP sets in the analysis of the real data set.**

As shown in Figure 1, the estimated accuracies were highly sensitive to the choice of *b* and, for SIS10K and RAND10K, deteriorated with *b* approaching 0 and *b*>0.015. In contrast, both RAND1K and SIS1K exhibited the best accuracies for *b*>0.015, showing horizontally asymptotic-like behaviour for increasing values of *b*. The accuracies were quite similar for RAND10K and SIS10K, except for *b* ranging between 0.015 and 0.03, where SIS10K yielded higher accuracies. In all cases, RAND1K had lower accuracies than SIS1K. As mentioned above, the higher accuracies for SIS may be, at least partially, due to the over-estimation induced during pre-selection by SIS.

For all values of *p*_0_, the accuracy showed very similar behaviour when *b* was varied. For both RAND10K and SIS10K, accuracies were best for *b* ranging between 0.005 and 0.012 and deteriorated when *b* tended towards 0 and also for increasing values of *b*. In contrast, both RAND1K and SIS1K exhibited the best accuracies for *b*>0.015, showing horizontally asymptotic-like behaviour for increasing values of *b*. In all cases, RAND1K had lower accuracies than SIS1K.

For both RAND10K and SIS10K, the least biased estimates, i.e. with regression coefficients closest to 1, were obtained for *b* between 0.002 and 0.008 and deteriorated down to 0.2 for increasing values of *b*. For RAND1K and SIS1K, the bias was more stable with respect to *b*, with the exception of a considerable bias upward for *p*_0_=0.9999 and *b* between 0.006 and 0.015.

Tables 5a, 5b, 5c and 5d show the estimates of accuracy and bias for the four SNP sets and the 16 prior specifications of the hyper-parameter pair (*p*_0_,*b*), as well as the combined estimates obtained from averaging GEBV across the prior specifications. In contrast to the observations made in the analysis of the QTLMAS XII data, averaging GEBV did not consistently improve the accuracies of single prior specifications. Whereas the best single estimate for SIS10K was 0.62 and the estimate combined across all 16 prior specifications was also 0.62, at least one single estimate in each of the three other SNP sets was slightly superior to the corresponding estimate combined from 16 prior specifications.

**Table 5:** **Accuracy estimates (bias estimates in brackets) for the four SNP sets (SIS10K, RAND10K, SIS1K, RAND1K) under 16 different prior specifications (pairs of (*p***_**0**_**, *b *)) and combined estimates across prior specifications (real data)**

**a - SIS10K**					
	*b*				
*p*_0_	0.004	0.008	0.012	0.016	Combined
0.9999	0.56 (1.18)	0.62 (1.03)	0.61 (0.83)	0.57 (0.63)	0.62 (0.95)
0.975	0.56 (0.98)	0.62 (1.00)	0.61 (0.83)	0.57 (0.63)	0.62 (0.92)
0.95	0.57 (0.96)	0.62 (0.98)	0.61 (0.83)	0.56 (0.63)	0.62 (0.91)
0.90	0.57 (0.91)	0.62 (0.97)	0.61 (0.82)	0.56 (0.63)	0.62 (0.89)
Combined	0.58 (1.06)	0.62 (1.00)	0.61 (0.83)	0.56 (0.63)	0.62 (0.92)
**b - RAND10K**					
	*b*				
*p*_0_	0.004	0.008	0.012	0.016	Combined
0.9999	0.56 (1.18)	0.63 (1.05)	0.60 (0.81)	0.52 (0.51)	0.61 (0.92)
0.975	0.57 (0.97)	0.62 (1.01)	0.61 (0.81)	0.52 (0.51)	0.61 (0.88)
0.95	0.57 (0.93)	0.61 (0.98)	0.61 (0.81)	0.52 (0.51)	0.61 (0.87)
0.90	0.57 (0.90)	0.61 (0.95)	0.61 (0.80)	0.52 (0.51)	0.61 (0.85)
Combined	0.58 (1.04)	0.62 (1.01)	0.61 (0.81)	0.52 (0.51)	0.61 (0.88)
**c - SIS1K**					
	*b*				
*p*_0_	0.004	0.008	0.012	0.016	Combined
0.9999	0.48 (1.05)	0.51 (1.16)	0.54 (1.14)	0.56 (1.08)	0.54 (1.16)
0.975	0.48 (0.96)	0.51 (1.02)	0.53 (1.07)	0.56 (1.08)	0.53 (1.08)
0.95	0.48 (0.94)	0.51 (1.02)	0.54 (1.05)	0.55 (1.08)	0.53 (1.07)
0.90	0.49 (0.94)	0.51 (0.99)	0.54 (1.03)	0.55 (1.03)	0.53 (1.04)
Combined	0.49 (1.00)	0.52 (1.08)	0.54 (1.09)	0.56 (1.07)	0.54 (1.10)
**d - RAND1K**					
	*b*				
*p*_0_	0.004	0.008	0.012	0.016	Combined
0.9999	0.34 (1.03)	0.46 (1.51)	0.50 (1.37)	0.52 (1.20)	0.49 (1.42)
0.975	0.38 (0.85)	0.42 (1.00)	0.48 (1.17)	0.51 (1.13)	0.47 (1.13)
0.95	0.40 (0.86)	0.42 (0.95)	0.48 (1.11)	0.51 (1.12)	0.47 (1.09)
0.90	0.42 (0.87)	0.44 (0.94)	0.48 (1.06)	0.51 (1.08)	0.48 (1.05)
Combined	0.41 (0.98)	0.44 (1.12)	0.49 (1.21)	0.52 (1.15)	0.48 (1.18)

## Discussion

In this article, we successfully applied MU, a shrinkage-based Bayesian variable selection that we had previously presented in [14], to the well studied and publicly available QTLMAS XII and real data sets with genome-wide marker coverage. In particular, we focussed our attention on comparing the impact of different prior specifications on the stability of QTL detection for genetic association and the stability of breeding value prediction for genomic selection. A Gibbs sampler for MCMC simulation and a GEM algorithm for MAP point estimation were implemented as C extensions to the software package R [22]. The source codes are publicly available as supporting information [see Additional files 2 and 3]. The computation time required by the implementations on a desktop PC appears feasible, being maximally a few hours for MCMC and a few minutes for GEM.

We have compared our results regarding QTL detection and false positive signals to findings from previous studies of the QTLMAS XII data. Overall, our analyses by MU ranked well among the association and mapping methods that were summarized by Crooks et al. [18]. Only one method [41] clearly outperformed MU. Instead of single SNP, this method exploited haplotype information of multiple SNP. Arguably, integration of this additional information into the regression model via a revised genotype matrix could improve the performance of MU.

Especially in the context of QTL detection, the collinearity of the putative predictors (SNP) in genome-wide dense marker data may cause problems in multi-locus models that assume mutual independence of predictors *a priori*, such as scattering of QTL signals over several markers. Several authors have suggested procedures to improve model performance in such settings: for example removing part of the data to reduce the collinearity (e.g. [46]). This general problem of applying MU and other Bayesian variable selection or shrinkage methods needs further research to improve their performance in QTL mapping.

The rather strong positive correlations among Bayes factors with QTL signals observed in several MCMC chains suggest an appreciable robustness of the results with regard to QTL detection under varying prior assumptions. Besides its advantages (see e.g. [27,47-50]) over such measures as *P*-values, the systematic differences in magnitude, that we observed between the Bayes factors from different MCMC chains, demonstrate the problems and limitations of the categories suggested by Jeffreys [34] and Kass and Raftery [32]. Specifically, blind application of decision rules based on these categories to declare positive QTL signals in genetic association and QTL mapping studies seem inadvisable. We stress the importance of an exhaustive analysis under varying prior assumptions. This need for a wide-ranging analysis is specifically evident, because, in general, weak prior knowledge exists on relevant biological parameters such as the prior probability of a positive QTL signal and the shape of the prior distributions for genetic effects. We tried to alleviate this problem by combining Bayes factor information from several analyses under varying prior specifications.

Obviously, an exhaustive sensitivity analysis under varying prior assumptions is necessary in shrinkage-based or other Bayesian variable selection approaches in general. However, we argue that MU provides some solution to the open problem of selecting relevant variables in Bayesian shrinkage approaches (see [17]), because MU provides a formal framework for hypothesis testing and consequently for the calculation of Bayes factors, in contrast to most other shrinkage approaches.

For the purpose of genomic prediction, MU was competitive with other studies in the estimation of GEBV for both data sets. For the simulated QTLMAS XII data set, it is noteworthy that we considered only four prior specifications in our analysis and did not attempt an exhaustive coverage of the hyper-parameter space. For this data set, our main focus was to compare MCMC and GEM estimations. Although point estimation via the GEM algorithm produced accuracies of GEBV that were inferior to accuracies from point estimation by MCMC for the single prior specifications, accuracy for GEM estimation was improved by combining GEBV across prior specifications to almost the same level as MCMC results.

In the analysis of the real data set, we explored a larger part of the hyper-parameter space and considered a dense grid of hyper-parameter values. Thus, we were able to assess accuracies of GEBV and differences more comprehensively than for the QTLMAS XII data set. Our results showed that the estimated accuracies were very sensitive to the choice of the hyper-parameter *b* in MU and that the sensitivity increased with the number of markers. Unfortunately, comparison of the sensitivity with other Bayesian genomic prediction approaches was hampered, because accuracies from an extensive search across varying prior specifications in other approaches are not documented for this data set.

In very poorly stated problems with many more SNP than individuals, as in the real data set, it may be beneficial to decrease the number of SNP to reduce this disparity prior to variable selection [38,51]. This will save computer storage capacity and may provide better convergence properties for the algorithms. In this study, we compared random sampling of SNP with sure independence screening (SIS) [38] as two simple methods of SNP pre-selection. For SIS, we followed the pre-selection and cross-validation procedure by [25] to be able to compare MU with the Bayesian genomic prediction methods considered in that study. Our results suggest that MU is competitive with other approaches and SIS produces superior accuracies for GEBV on large parts of the hyper-parameter space, although random sampling and SIS produce almost identical accuracies in the case of 10 000 SNP under optimally chosen hyper-parameters. However, the absolute level of SIS accuracy estimates reported here may be biased upward, because SIS pre-selection depended not only on the training sets but also on the validation sets. This bias may hamper the comparison of SIS and random sampling results.

Pre-selection of SNP surely remains an issue for future research and SIS cannot be the final solution, since some drawbacks of SIS remain unresolved. Because SIS exploits only marginal correlations between markers and the phenotype, and LD between markers is ignored, this approach is associated with a risk that too many SNP in the proximity of a QTL, that carry essentially identical information, are pre-selected. In contrast, SNP with a low marginal correlation but still in LD with a QTL have no chance of entering the set of pre-selected SNP. Methods that simultaneously exploit the connection between SNP and the phenotype and the LD structure between markers could be more appropriate.

Three approaches are available for choosing hyper-parameters. First, cross-validation can be employed to detect the optimal prior configuration with respect to the assessment of prediction for some specific set of individuals. This approach is probably the most suitable and most widely used approach for prediction purposes in experimental studies.

Second, prior expectation about the heritability and limiting assumptions about the genetic architecture of the trait yield a criterion to calibrate hyper-parameters via the prior variance of additive genetic effect sizes. In this study, we derived such a criterion for MU and tested its performance in the analysis of the real data. However, the results do not support the idea that values of hyper-parameters corresponding to realistic heritabilities according to this criterion positively affect prediction accuracy.

As a third alternative to choose hyper-parameters, expert knowledge may be available on the size of hyper-parameters. However, these three approaches are not free of error in practical situations and, therefore, doubt will remain for any specific prior choice. Combining results from varying prior specifications using ”poor man’s” model averaging, as was done here, may provide some solution, as a wider choice of hyper-parameters can be integrated into ”consensus” estimates. For this purpose, an approach like MU is especially suitable, because it comprises a wide and flexible family of prior distributions.

To reduce the problem induced by the sensitivity to the choice of hyper-parameters, it is common practice in Bayesian modeling to add an extra layer to the hierarchy and to assign own prior distributions to at least some of the hyper-parameters. This is commonly done, for example, in Bayesian LASSO and stochastic search variable selection methods such as BayesCn and BayesDn (see e.g. [52]). We have refrained from doing so in this study, for the following reasons: (1) the sensitivity problem may just be moved to the next layer of the hierarchy and the method may then become sensitive to the parameters controlling the prior distribution of a hyper-parameter (see [25]), and (2) even if the approach may work in MCMC implementation, the hyper-parameter may not be identifiable in faster maximum *a posteriori* estimation algorithms such as EM, GEM or variational Bayes algorithms (e.g., [16,25]). This behaviour would possibly have a negative impact on the performance of the GEM estimation algorithm that was introduced in this study to make the MU method scalable for large SNP panels with thousands of variables and individuals. Finally, assigning priors to hyper-parameters may result in bad separation of QTL signals [5], which is less important in genomic prediction but of major concern in genetic association studies.

## Competing interests

The authors declare that they have no competing interests.

## Authors’ contributions

All authors were involved in the conception and the design of the study. TK derived the fully conditional distributions for the Gibbs sampler and the GEM algorithm of MU, implemented the C modules, performed the data analysis and drafted the manuscript. All authors participated in the interpretation of results. EL and MJS critically revised the manuscript. All authors read and approved the final manuscript.

## Supplementary Material

Additional file 1 **Complete model specifications and fully conditional distributions.** Complete distributional specification of the likelihood and of the priors in MU, derivation of the fully conditional distributions for the Gibbs sampler and their expected values for GEM. Click here for file

Additional file 2 **C code for the Gibbs sampler.** The C implementation of the Gibbs sampler. Click here for file

Additional file 3 **C code for the GEM algorithm.** The C implementation of the generalized expectation-maximization algorithm (GEM). Click here for file
