# Supplementary material for: Impact of prior specifications in ashrinkage-inducing Bayesian model for quantitative trait mapping and genomic prediction
Source: Genet Sel Evol. 2013 Jul 8;45(1):24. doi: 10.1186/1297-9686-45-24 (PMC3750442; doi:10.1186/1297-9686-45-24)
Supplement: Additional file 1 — Complete model specifications and fully conditional distributions. Complete distributional specification of the likelihood and of the priors in MU, derivation of the fully conditional distributions for the Gibbs sampler and their expected values for GEM. [file 1297-9686-45-24-S1.pdf]

Complete distributional specification of the likelihood as well as of the priors in MU, derivation of the fully conditional distributions for the Gibbs sampler and their expected values for GEM.

First, we present a generic formula for the posterior distribution of the model parameters in MU. To simplify the notation, let $Y = (Y_{kj})$ be the vector of the phenotype observations and $\mathbf{X} = (x_{kjm})$ the $N \times M$-matrix of the genotype observations ($k = 1, \ldots, K$; $j = 1, \ldots, N_k$; $m = 1, \ldots, M$; $N = \sum_{k=1}^{K} N_k$). Further, we denote the vector of all model parameters by

$$\Theta = (\alpha, \beta_1, \ldots, \beta_M, u_1, \ldots, u_K, \sigma_u^2, \sigma^2)$$

with support $S = \mathbb{R} \times [-l, l]^M \times R^K \times R_+ \times R_+$, where $l > 0$.

We use the generic notation $\Theta_{-\theta}$ to indicate the vector of all model parameters except the univariate parameter θ. Mutual independence is assumed for all univariate random variables in $\Theta_{-\sigma_u^2}$ conditionally given σ_u^2, and all parameters except $u_1, \ldots, u_K$ are assumed independent of σ_u^2.

According to Bayes' theorem, the joint posterior distribution of Θ can then be expressed up to a multiplicative constant as

$$\pi(\Theta|Y, \mathbf{X}) \propto L(Y|\mathbf{X}, \Theta) p(\alpha) \prod_{m=1}^{M} p(\beta_m) \prod_{k=1}^{K} p(u_k|\sigma_u^2) p(\sigma_u^2) p(\sigma^2), \qquad (1)$$

where $\pi(\cdot)$ is the probability density function (pdf) of the joint posterior and in the following also the pdf of any fully conditional posterior, $L(\cdot)$ the likelihood function, wheras $p(\cdot)$ denotes a univariate prior pdf.

Likelihood

Assuming independent and identically normally distributed error terms (ϵ_{kj}), the likelihood of the parameter vector Θ can be expressed as

$$L(Y|\mathbf{X}, \Theta) \propto \sigma^{-N} \exp\left(-\frac{1}{2\sigma^2} \sum_{k=1}^{K} \sum_{j=1}^{N_k} \left[Y_{kj} - \alpha - \sum_{m=1}^{M} \beta_m X_{kjm} - u_k \right]^2 \right). \qquad (2)$$

In the following, we will list the specification of the prior distribution, derive the univariate fully conditional posterior pdf for each model parameter and provide the corresponding expected value. Except for the effect size parameters (β_m) in MU, the choice of prior is conjugate for all univariate fully conditional posteriors. Note that in the following $L(Y|\mathbf{X}, \theta)$ is the univariate likelihood function of the parameter θ obtained from (2) by omitting a multiplicative constant involving only elements of $\Theta_{-\theta}$

Common intercept α

Assigning a normally distributed prior with mean 0 and variance $c > 0$ to α, its fully conditional posterior pdf is

$$\begin{aligned}\pi(\alpha|Y,\mathbf{X},\Theta_{-\alpha}) &\propto L(Y|\mathbf{X},\alpha)p(\alpha)\\ &\propto \exp\left(-\frac{1}{2\sigma^2}\left[N\alpha^2 - 2\alpha\sum_{k=1}^{K}\sum_{j=1}^{N_k} R_{kj}^{-\alpha}\right]\right)\exp\left(-\frac{\alpha^2}{2c}\right)\\ &\propto \exp\left(-\frac{1}{2}\left(\frac{c\sigma^2}{Nc+\sigma^2}\right)^{-1}\left[\alpha - \frac{c\sigma^2}{Nc+\sigma^2}\cdot\frac{1}{\sigma^2}\sum_{k=1}^{K}\sum_{j=1}^{N_k} R_{kj}^{-\alpha}\right]^2\right),\end{aligned} \tag{3}$$

where $R_{kj}^{-\alpha} = Y_{kj} - \sum_{m=1}^{M}\beta_m X_{kjm} - u_k$. The fully conditional posterior distribution of α is therefore Gaussian with expected value m_α and variance v_α, where

$$m_\alpha = v_\alpha\sigma^{-2}\sum_{k=1}^{K}\sum_{j=1}^{N_k} R_{kj}^{-\alpha} \text{ and } v_\alpha = c\sigma^2\left(Nc+\sigma^2\right)^{-1}.$$

Effect size parameters β_m ($m = 1, \ldots, M$) in MU

Let $\varphi(x)$ denote the pdf of the univariate standard normal distribution and $\Phi(x)$ its cumulative distribution function. The pdf of the fully conditional posterior of β_m is

$$\begin{aligned}\pi(\beta_m|Y,\mathbf{X},\Theta_{-\beta_m}) &\propto L(Y|\mathbf{X},\beta_m)p(\beta_m)\\ &\propto \exp\left(-\frac{1}{2\sigma^2}\left[\beta_m^2\sum_{k=1}^{K}\sum_{j=1}^{N_k} X_{kjm}^2 - 2\beta_m\sum_{k=1}^{K}\sum_{j=1}^{N_k} X_{kjm}R_{kj}^{-\beta_m}\right]\right)p(\beta_m)\\ &\propto \exp\left(-\frac{1}{2}\left[\frac{\beta_m - m_{\beta_m}}{\sqrt{v_{\beta_m}}}\right]^2\right)p(\beta_m)\\ &\propto \frac{1}{\sqrt{v_{\beta_m}}}\varphi\left(\frac{\beta_m - m_{\beta_m}}{\sqrt{v_{\beta_m}}}\right)p(\beta_m),\end{aligned} \tag{4}$$

where

$$R_{kj}^{-\beta_m} = Y_{kj} - \alpha - \sum_{\substack{m'=1\\ m'\neq m}}^{M}\beta_{m'}X_{kjm'} - u_k,$$

$$m_{\beta_m} = \sum_{k=1}^{K}\sum_{j=1}^{N_k} X_{kjm}R_{kj}^{-\beta_m}\left(\sum_{k=1}^{K}\sum_{j=1}^{N_k} X_{kjm}^2\right)^{-1} \text{ and } v_{\beta_m} = \sigma^2\left(\sum_{k=1}^{K}\sum_{j=1}^{N_k} X_{kjm}^2\right)^{-1}.$$

As the prior pdf of β_m is a step function, namely

$$p(\beta_m) = q_{\text{neg}}I_{[-l,-b]}(\beta_m) + q_0 I_{(-b,b)}(\beta_m) + q_{\text{pos}}I_{[b,l]}(\beta_m), \tag{5}$$

where $0 < b < l$, $q_{\text{neg}} = q_{\text{pos}} = (1 - p_0)[2(l - b)]^{-1}$ with $p_0 \in (0, 1)$, and $q_0 = p_0(2b)^{-1}$, the fully conditional posterior distribution of β_m is a discrete mixture of three truncated normal distributions. The three mixture probabilities, say $c_{\text{neg}} = P(\beta_m \in [-l, -b]|Y, \mathbf{X}, \Theta_{-\beta_m})$, $c_0 = P(\beta_m \in [-b, b]|Y, \mathbf{X}, \Theta_{-\beta_m})$ and $c_{\text{pos}} = P(\beta_m \in [b, l]|Y, \mathbf{X}, \Theta_{-\beta_m})$ are obtained by first calculating suitable weights, say $d_{\text{neg}}, d_0, d_{\text{pos}}$:

$$\begin{aligned} d_{\text{neg}} &= q_{\text{neg}} \int_{-l}^{-b} \frac{1}{\sqrt{v_{\beta_m}}} \varphi\left(\frac{\beta_m - m_{\beta_m}}{\sqrt{v_{\beta_m}}}\right) d\beta_m \\ &= q_{\text{neg}} \left[\Phi\left(\frac{-b - m_{\beta_m}}{\sqrt{v_{\beta_m}}}\right) - \Phi\left(\frac{-l - m_{\beta_m}}{\sqrt{v_{\beta_m}}}\right)\right], \end{aligned} \tag{6}$$

similarly

$$d_0 = q_0 \left[\Phi\left(\frac{b - m_{\beta_m}}{\sqrt{v_{\beta_m}}}\right) - \Phi\left(\frac{-b - m_{\beta_m}}{\sqrt{v_{\beta_m}}}\right)\right] \tag{7}$$

and

$$d_{\text{pos}} = q_{\text{pos}} \left[\Phi\left(\frac{l - m_{\beta_m}}{\sqrt{v_{\beta_m}}}\right) - \Phi\left(\frac{b - m_{\beta_m}}{\sqrt{v_{\beta_m}}}\right)\right]. \tag{8}$$

Each of the weights is then divided by their sum $D = d_{\text{neg}} + d_0 + d_{\text{pos}}$ to obtain the mixture probabilities, *i.e.* $c_{\text{neg}} = d_{\text{neg}}/D$, $c_0 = d_0/D$ and $c_{\text{pos}} = d_{\text{pos}}/D$.

The expected value of this distribution is the weighted sum $c_{\text{neg}}m_{\text{neg}} + c_0 m_0 + c_{\text{pos}}m_{\text{pos}}$, where

$$m_{\text{neg}} = m_{\beta_m} + \sqrt{v_{\beta_m}} \left[\phi\left(\frac{-l - m_{\beta_m}}{v_{\beta_m}}\right) - \phi\left(\frac{-b - m_{\beta_m}}{v_{\beta_m}}\right)\right] / \left[\Phi\left(\frac{-b - m_{\beta_m}}{\sqrt{v_{\beta_m}}}\right) - \Phi\left(\frac{-l - m_{\beta_m}}{\sqrt{v_{\beta_m}}}\right)\right],$$

$$m_{\text{neg}} = m_{\beta_m} + \sqrt{v_{\beta_m}} \left[\phi\left(\frac{-b - m_{\beta_m}}{v_{\beta_m}}\right) - \phi\left(\frac{b - m_{\beta_m}}{v_{\beta_m}}\right)\right] / \left[\Phi\left(\frac{b - m_{\beta_m}}{\sqrt{v_{\beta_m}}}\right) - \Phi\left(\frac{-b - m_{\beta_m}}{\sqrt{v_{\beta_m}}}\right)\right],$$

$$m_{\text{neg}} = m_{\beta_m} + \sqrt{v_{\beta_m}} \left[\phi\left(\frac{b - m_{\beta_m}}{v_{\beta_m}}\right) - \phi\left(\frac{l - m_{\beta_m}}{v_{\beta_m}}\right)\right] / \left[\Phi\left(\frac{l - m_{\beta_m}}{\sqrt{v_{\beta_m}}}\right) - \Phi\left(\frac{b - m_{\beta_m}}{\sqrt{v_{\beta_m}}}\right)\right].$$

Polygenic effects u_k ($k = 1, \ldots, K$)

Assigning a normally distributed prior with mean 0 and variance σ_u^2 to u_k, its fully conditional posterior pdf is

$$\begin{aligned} \pi(u_k|Y, \mathbf{X}, \Theta_{-u_k}) &\propto L(Y|\mathbf{X}, u_k)p(u_k|\sigma_u^2) \\ &\propto \exp\left(-\frac{1}{2\sigma^2}\left[N_k u_k^2 - 2u_k \sum_{j=1}^{N_k} R_{kj}^{-u_k}\right]\right) \exp\left(-\frac{u_k^2}{2\sigma_u^2}\right) \\ &\propto \exp\left(-\frac{1}{2}\left(\frac{\sigma_u^2\sigma^2}{N_k\sigma_u^2 + \sigma^2}\right)^{-1}\left[u_k - \frac{\sigma_u^2\sigma^2}{N_k\sigma_u^2 + \sigma^2} \cdot \frac{1}{\sigma^2} \cdot \sum_{j=1}^{N_k} R_{kj}^{-u_k}\right]^2\right), \end{aligned} \tag{9}$$

where $R_{kj}^{-u_k} = Y_{kj} - \alpha - \sum_{m=1}^{M} \beta_m X_{kjm}$. The fully conditional posterior distribution of u_k is therefore Gaussian with expected value m_{u_k} and variance v_{u_k}, where

$$m_{u_k} = v_{u_k} \sigma^{-2} \sum_{j=1}^{N_k} R_{kj}^{-u_k} \text{ and } v_{u_k} = \sigma_u^2 \sigma^2 \left(N_k \sigma_u^2 + \sigma^2 \right)^{-1}.$$

Between-families variance component σ_u^2

Assigning an inverse-gamma distribution with shape parameter $s_u > 0$ and rate parameter $r_u > 0$ as the prior of σ_u^2, its fully conditional posterior pdf is

$$\begin{aligned} \pi(\sigma_u^2 | Y, \mathbf{X}, \Theta_{-\sigma_u^2}) &\propto \prod_{k=1}^{K} p(u_k | \sigma_u^2) p(\sigma_u^2) \\ &\propto \left(\sigma_u^2\right)^{-K/2} \exp\left(-\frac{1}{2\sigma_u^2} \sum_{k=1}^{K} u_k^2 \right) \left(\sigma_u^2\right)^{-s_u - 1} \exp\left(-\frac{r_u}{\sigma_u^2} \right) \\ &\propto \left(\sigma_u^2\right)^{-(K/2 + s_u) - 1} \exp\left(-\frac{1}{\sigma_u^2} \left[\frac{1}{2} \sum_{k=1}^{K} u_k^2 + r_u \right] \right) \end{aligned} \tag{10}$$

The fully conditional posterior distribution of σ_u^2 is therefore inverse-gamma with the shape parameter $K/2 + s_u$ and the rate parameter $\sum_{k=1}^{K} u_k^2/2 + r_u$. The expected value of this distribution is $\left(\sum_{k=1}^{K} u_k^2/2 + r_u \right) / (K/2 + s_u - 1)$.

Residual variance σ^2

Assigning an inverse-gamma distribution with shape parameter $s > 0$ and rate parameter $r > 0$ as the prior of σ^2, its fully conditional posterior pdf is

$$\begin{aligned} \pi(\sigma^2 | Y, \mathbf{X}, \Theta_{-\sigma^2}) &\propto L(Y | \mathbf{X}, \sigma^2) p(\sigma^2) \\ &\propto \left(\sigma^2\right)^{-N/2} \exp\left(-\frac{1}{2\sigma^2} \sum_{k=1}^{K} \sum_{j=1}^{N_k} R_{kj}^2 \right) \left(\sigma^2\right)^{-s-1} \exp\left(-\frac{r}{\sigma^2} \right) \\ &\propto \left(\sigma^2\right)^{-(N/2 + s) - 1} \exp\left(-\frac{1}{\sigma^2} \left[\frac{1}{2} \sum_{k=1}^{K} \sum_{j=1}^{N_k} R_{kj}^2 + r \right] \right), \end{aligned} \tag{11}$$

where $R_{kj} = Y_{kj} - \alpha - \sum_{m=1}^{M} \beta_m X_{kjm} - u_k$. The fully conditional posterior distribution of σ^2 is therefore inverse-gamma with the shape parameter $N/2 + s$ and the rate parameter $\sum_{k=1}^{K} \sum_{j=1}^{N_k} R_{kj}^2/2 + r$. The expected

value of this distribution is $\left(\sum_{k=1}^{K}\sum_{j=1}^{N_k} R_{kj}^2/2 + r\right)/(N/2 + s - 1)$.
