# Supplementary material for: Impact of prior specifications in ashrinkage-inducing Bayesian model for quantitative trait mapping and genomic prediction
Source: Genet Sel Evol. 2013 Jul 8;45(1):24. doi: 10.1186/1297-9686-45-24 (PMC3750442; doi:10.1186/1297-9686-45-24)
Supplement: Additional file 2 — C code for the Gibbs sampler. The C implementation of the Gibbs sampler. [file 1297-9686-45-24-S2.pdf]

The C implementation of the Gibbs sampler

We used the C model below under a Windows operating system. In this case, the C code below has to be compiled first into a dynamic link library with .dll extension (see R documentation for details). This dll is then be loaded by R with the command:

```
> dyn.load("gibbs.dll")
```

An example call in R is

```
> RESULT <- .Call("gibbs", y, X, famID, M, N, K, alpha, beta, u, sigma2, sigma2u,
                  borders, pp, c, shape, rate, B, T )
```

Here, the arguments $y, ..., T$ refer to appropriate R objects. The result is stored in a list object $RESULT$.

```
#include <R.h>
#include <Rinternals.h>
#include <Rmath.h>
#include <math.h>

void alphaupdate(double *,double *, double , int , double );
void varupdate(double *, double *, int ,double,double );
void uupdate(double *, double *, int *, double , double, int, int );
void betaupdate(double *, double *, double *, double , double *,
                double , double , double , int , int );

SEXP gibbs(SEXP yIN, /* phenotype */
           SEXP xmatIN, /* genotype matrix */
           SEXP famIDIN, /* vector with family indicators */

           SEXP MIN, /* number of markers */
           SEXP NIN, /* number of individuals */
           SEXP KIN, /* number of families */

           SEXP alphaIN, /* initial values */
           SEXP betaIN,
           SEXP uIN,
           SEXP sigma2IN,
           SEXP sigma2uIN,

           SEXP bordersIN, /* parameters (-l, -b, b, l) in MU */
           SEXP ppIN,   /* vector ( (1-p0)/2, p0, (1-p0)/2 ) */
           SEXP cIN,    /* prior variance of alpha */
           SEXP shapeIN, /* prior shape for variance components */
           SEXP rateIN, /* prior rate for variance components */

           SEXP BIN, /* number of iterations */
           SEXP TIN  /* size of thinning */
           )
{

SEXP ans, alphaAns, sigma2Ans, sigma2uAns, uAns, betaAns, resAns;

double alphaCur=REAL(alphaIN)[0],
       sigma2Cur=REAL(sigma2IN)[0],
       sigma2uCur=REAL(sigma2uIN)[0],
       *resids,
```

```
       *betaCur,
       *uCur;

int *famID;

int N = INTEGER(NIN)[0],
    M = INTEGER(MIN)[0],
    B = INTEGER(BIN)[0],
    K = INTEGER(KIN)[0],
    T = INTEGER(TIN)[0],
    k, m, n, b, t;

double c = REAL(cIN)[0],
       shape = REAL(shapeIN)[0],
       rate = REAL(rateIN)[0],
       qnull  = REAL(ppIN)[1]/(REAL(bordersIN)[2]-REAL(bordersIN)[1]),
       qminus = REAL(ppIN)[0]/(REAL(bordersIN)[1]-REAL(bordersIN)[0]),
       qplus  = REAL(ppIN)[2]/(REAL(bordersIN)[3]-REAL(bordersIN)[2]);

uCur = (double *)             calloc(K, sizeof(double));
for ( k = 0; k < K; k++) uCur[k]=REAL(uIN)[k];
betaCur = (double *)          calloc(M, sizeof(double));
for ( m = 0; m < M; m++) betaCur[m]=REAL(betaIN)[m];
resids = (double *)           calloc(N, sizeof(double));
famID = (int *)           calloc(N, sizeof(int));
for (n=0; n < N ; n++){
   famID[n]=INTEGER(famIDIN)[n];
   resids[n]=REAL(yIN)[n] - alphaCur - uCur[famID[n]-1];
   for (m = 0; m < M; m++)
      resids[n] -= betaCur[m]*REAL(xmatIN)[n+m*N];
}
PROTECT( alphaAns = allocVector(REALSXP, B));
PROTECT( sigma2Ans = allocVector(REALSXP, B));
PROTECT( sigma2uAns = allocVector(REALSXP, B));
PROTECT( uAns = allocMatrix(REALSXP, B, K));
PROTECT( betaAns = allocMatrix(REALSXP, B, M));
PROTECT( resAns = allocMatrix(REALSXP, B, N));

GetRNGstate();

for (b=0; b < B; b++){
   for (t=0; t < T; t++){
      alphaupdate(&alphaCur, resids, c, N, sigma2Cur);
      varupdate(&sigma2Cur, resids, N, shape, rate);
      varupdate(&sigma2uCur, uCur, K, shape, rate);
      uupdate(uCur, resids, famID, sigma2Cur, sigma2uCur, N, K);
      betaupdate(betaCur, resids, REAL(xmatIN), sigma2Cur, REAL(bordersIN),
                 qminus, qnull, qplus, M, N);
   }
   for (m = 0; m < M; m++) REAL(betaAns)[b+m*B] = betaCur[m];
   for (k = 0; k < K; k++) REAL(uAns)[b+k*B] = uCur[k];
   REAL(alphaAns)[b] = alphaCur;
   REAL(sigma2Ans)[b] = sigma2Cur;
   REAL(sigma2uAns)[b] = sigma2uCur;
   for (n =0; n < N; n++) REAL(resAns)[b+n*B] = resids[n];
}

PutRNGstate();
PROTECT(ans = allocVector(VECSXP,6));
SET_VECTOR_ELT(ans, 0, betaAns);
SET_VECTOR_ELT(ans, 1, uAns);
SET_VECTOR_ELT(ans, 2, alphaAns);
SET_VECTOR_ELT(ans, 3, sigma2Ans);
SET_VECTOR_ELT(ans, 4, sigma2uAns);
```

```
SET_VECTOR_ELT(ans, 5, resAns);

free(uCur);
free(resids);
free(betaCur);
UNPROTECT(7);
return(ans);

}

void betaupdate(double *beta, double *res, double *x,
                double s2,
                double *borders,
                double qminus, double qnull, double qplus,
                int M, int N
                )
{
   int m, n;
   double mu, sd, p1, p2, p3, p4,
          cminus, cnull, cplus;

   for (m = 0; m < M; m++){
      mu=0.0;
      sd=0.0;
      for (n = 0; n < N; n++){
          res[n] += x[n+m*N]*beta[m];
          mu += x[n+m*N]*res[n];
          sd += x[n+m*N]*x[n+m*N];
      }
      mu = mu/sd;
      sd = sqrt(s2/sd);
      p1 = pnorm(borders[0], mu, sd, 1, 0);
      p2 = pnorm(borders[1], mu, sd, 1, 0);
      p3 = pnorm(borders[2], mu, sd, 1, 0);
      p4 = pnorm(borders[3], mu, sd, 1, 0);
      cminus = qminus*(p2-p1);
      cnull = qnull*(p3-p2);
      cplus = qplus*(p4-p3);

      if ( rbinom(1.0, cnull/(cminus+cnull+cplus) )==1.0 )
         beta[m] = mu + sd*qnorm( p2+unif_rand()*(p3-p2), 0.0, 1.0, 1, 0 );
      else if ( rbinom(1.0, cminus/(cminus + cplus) )==1.0 )
         beta[m] = mu + sd*qnorm( p1+unif_rand()*(p2-p1), 0.0, 1.0, 1, 0 );
      else
         beta[m] = mu + sd*qnorm( p3+unif_rand()*(p4-p3), 0.0, 1.0, 1, 0 );

      for (n = 0; n < N; n++)
          res[n] -= x[n+m*N]*beta[m];
   }

}

void uupdate(double *u,
             double *resids,
             int *famID,
             double s2,
             double s2u,
             int N,
             int K
             ){
   double *means, *vars;
   int n, k;
   means = (double *) calloc(K, sizeof(double));
   vars = (double *) calloc(K, sizeof(double));
```

```
   for ( k = 0; k < K; k++){
       means[k]=0.0;
       vars[k]=0.0;
   }
   for (n=0; n < N; n++){
      resids[n] += u[famID[n]-1];
      vars[famID[n]-1] += 1.0;
      means[famID[n]-1] += resids[n];
   }
   for ( k = 0; k < K; k++){
       vars[k] = s2*s2u/( s2u*vars[k] + s2);
       means[k] = vars[k]*means[k]/s2;
       u[k] = rnorm(means[k],sqrt(vars[k]));

   }
   free(means);
   free(vars);
   for (n=0; n < N; n++)
      resids[n] -= u[famID[n]-1];
}


void varupdate(double *sigma2,
               double *eff,
               int Neff,
               double s,
               double r){
   int i;
   double rate, shape;
   rate = 0;
   for (i = 0; i < Neff; i++)
      rate += eff[i]*eff[i];
   rate = r + 0.5*rate;
   shape = s + Neff/2.0;
   *sigma2 = 1/rgamma(shape, 1.0/rate);

}

void alphaupdate(double *alpha,
                 double *resids,
                 double c,
                 int N,
                 double sigma2){
   int n;
   double meana, vara;
   meana=0.0;

   for (n=0; n<N; n++ ){
      resids[n] += *alpha;
      meana += resids[n];
   }
   vara = c * sigma2/(N*c+ sigma2 );
   meana = vara * meana / sigma2;

   *alpha = rnorm(meana,sqrt(vara));

   for (n=0; n<N; n++ )
      resids[n] -= *alpha;
}
```
