# Supplementary material for: Impact of prior specifications in ashrinkage-inducing Bayesian model for quantitative trait mapping and genomic prediction
Source: Genet Sel Evol. 2013 Jul 8;45(1):24. doi: 10.1186/1297-9686-45-24 (PMC3750442; doi:10.1186/1297-9686-45-24)
Supplement: Additional file 3 — C code for the GEM algorithm. The C implementation of the generalized expectation-maximization algorithm (GEM). [file 1297-9686-45-24-S3.pdf]

The C implementation of the generalized expectation-maximization algorithm (GEM)

We used the C model below under a Windows operating system. In this case, the C code below has to be compiled first into a dynamic link library with .dll extension (see R documentation for details). This dll is then be loaded by R with the command:

```
> dyn.load("gem.dll")
```

An example call in R is

```
> RESULT <- .Call("gem", y, X, M, N, alpha, beta, sigma2,
                    borders, pp, c, shape, rate, prec)
```

Here, the arguments $y, ..., prec$ refer to appropriate R objects. The result is stored in a list object $RESULT$.

```
#include <R.h>
#include <Rinternals.h>
#include <Rmath.h>
#include <math.h>

void alphaupdate(double *,double *, double , int , double );
void varupdate(double *, double *, int ,double,double );
void betaupdate(double *, double *, double *, double , double *,
                double , double , double , int , int );

SEXP gem(SEXP yIN,         /* phenotypes */
            SEXP xmatIN,   /* genotype matrix */
            SEXP MIN,      /* number of SNPs */
            SEXP NIN,      /* number of individuals */

            SEXP alphaIN,  /* initial values */
            SEXP betaIN,
            SEXP sigma2IN,

           SEXP bordersIN, /* parameters (-l, -b, b, l) in MU */
           SEXP ppIN,      /* vector ( (1-p0)/2, p0, (1-p0)/2 ) */
           SEXP cIN,       /* prior variance of alpha */
           SEXP shapeIN,   /* prior shape for variance component */
           SEXP rateIN,    /* prior rate for variance component */

           SEXP precIN /* precision parameter for convergence check */
           )
{


SEXP ans, alphaAns, sigma2Ans,
     betaAns, countAns, resAns, gevAns;

double alphaCur=REAL(alphaIN)[0], oldalpha,
       sigma2Cur=REAL(sigma2IN)[0], oldsigma2,
       *betaCur, *oldbeta,
       *resids,
       pardiff, count, test;

int N = INTEGER(NIN)[0],
    M = INTEGER(MIN)[0],
    m, n;
```

```
double c = REAL(cIN)[0],
       shape = REAL(shapeIN)[0],
       rate = REAL(rateIN)[0],
       qnull  = REAL(ppIN)[1]/(REAL(bordersIN)[2]-REAL(bordersIN)[1]),
       qminus = REAL(ppIN)[0]/(REAL(bordersIN)[1]-REAL(bordersIN)[0]),
       qplus  = REAL(ppIN)[2]/(REAL(bordersIN)[3]-REAL(bordersIN)[2]),
       convprec = REAL(precIN)[0];

betaCur = (double *)          calloc(M, sizeof(double));
oldbeta = (double *)          calloc(M, sizeof(double));


for ( m = 0; m < M; m++) {
    betaCur[m]=REAL(betaIN)[m];
}
resids = (double *) calloc(N, sizeof(double));

for (n=0; n < N ; n++){
   resids[n]=REAL(yIN)[n] - alphaCur;
   for (m = 0; m < M; m++)
      resids[n] -= betaCur[m]*REAL(xmatIN)[n+m*N];
}
PROTECT( alphaAns = allocVector(REALSXP, 1));
PROTECT( sigma2Ans = allocVector(REALSXP, 1));
PROTECT( betaAns = allocVector(REALSXP,  M));
PROTECT( countAns = allocVector(REALSXP, 1));
PROTECT( resAns = allocVector(REALSXP, N));
PROTECT( gevAns = allocVector(REALSXP, N));


GetRNGstate();

test = 1.0;
count = 0.0;

while (test>0.0 && count < 1000.0){

      test = 0.0;
      pardiff = 0.0;

      oldalpha = alphaCur;
      alphaupdate(&alphaCur, resids, c, N, sigma2Cur);
      pardiff += fabs(oldalpha - alphaCur);

      oldsigma2 = sigma2Cur;
      varupdate(&sigma2Cur, resids, N, shape, rate);
      pardiff += fabs(oldsigma2 - sigma2Cur);

      for (m = 0; m < M; m++) oldbeta[m] = betaCur[m];
      betaupdate(betaCur, resids, REAL(xmatIN), sigma2Cur, REAL(bordersIN),
                 qminus, qnull, qplus, M, N);
      for (m = 0; m < M; m++) pardiff += fabs(oldbeta[m]-betaCur[m]);

      if( pardiff >= ( M   + 2)*convprec) test +=1.0;
      count += 1.0;

}

   for (m = 0; m < M; m++) REAL(betaAns)[m] = betaCur[m];
   for (n = 0; n < N; n++) {
       REAL(resAns)[n] = resids[n];
       REAL(gevAns)[n] = REAL(yIN)[n] - resids[n] - alphaCur;
   }
```

```
   REAL(alphaAns)[0] = alphaCur;
   REAL(sigma2Ans)[0] = sigma2Cur;
   REAL(countAns)[0] = count;

PutRNGstate();
PROTECT(ans = allocVector(VECSXP,6));
SET_VECTOR_ELT(ans, 0, betaAns);
SET_VECTOR_ELT(ans, 1, alphaAns);
SET_VECTOR_ELT(ans, 2, sigma2Ans);
SET_VECTOR_ELT(ans, 3, countAns);
SET_VECTOR_ELT(ans, 4, resAns);
SET_VECTOR_ELT(ans, 5, gevAns);

free(betaCur);
free(oldbeta);
free(resids);
UNPROTECT(7);
return(ans);
}


void betaupdate(double *beta, double *res, double *x,
                double s2,
                double *borders,
                double qminus, double qnull, double qplus,
                int M, int N
                )
{
   int m, n;
   double mu, sd, p1, p2, p3, p4, q1, q2, q3, q4,
          cminus, cnull, cplus, D,
          meannull, meanminus, meanplus;

   for (m = 0; m < M; m++){
      mu=0.0;
      sd=0.0;
      for (n = 0; n < N; n++){
          res[n] += x[n+m*N]*beta[m];
          mu += x[n+m*N]*res[n];
          sd += x[n+m*N]*x[n+m*N];
      }
      mu = mu/sd;
      sd = sqrt(s2/sd);
      p1 = pnorm(borders[0], mu, sd, 1, 0);
      p2 = pnorm(borders[1], mu, sd, 1, 0);
      p3 = pnorm(borders[2], mu, sd, 1, 0);
      p4 = pnorm(borders[3], mu, sd, 1, 0);
      cminus = qminus*(p2-p1);
      cnull = qnull*(p3-p2);
      cplus = qplus*(p4-p3);
      D = cminus + cnull + cplus;
      cminus = cminus/D;
      cnull = cnull/D;
      cplus = cplus/D;

      q1 = dnorm( (borders[0]-mu)/sd, 0.0, 1.0, 0);
      q2 = dnorm( (borders[1]-mu)/sd, 0.0, 1.0, 0);
      q3 = dnorm( (borders[2]-mu)/sd, 0.0, 1.0, 0);
      q4 = dnorm( (borders[3]-mu)/sd, 0.0, 1.0, 0);
      meanminus=0.0;
      meannull=0.0;
      meanplus=0.0;
      if (p2 - p1 > 0)   meanminus = mu + sd*( q1 - q2 )/(p2 - p1);
      if (p3 - p2 > 0)   meannull = mu  + sd*( q2 - q3 )/(p3 - p2);
```

```
      if (p4 - p3 > 0)   meanplus = mu  + sd*( q3 - q4 )/(p4 - p3);

      beta[m] = cminus*meanminus + cnull*meannull + cplus*meanplus;

      for (n = 0; n < N; n++)
          res[n] -= x[n+m*N]*beta[m];
   }
}


void varupdate(double *sigma2,
               double *eff,
               int Neff,
               double s,
               double r){
   int i;
   double rate, shape;
   rate = 0;
   for (i = 0; i < Neff; i++)
      rate += eff[i]*eff[i];
   rate = r + 0.5*rate;
   shape = s + Neff/2.0;
   *sigma2 = rate/(shape-1.0);
}

void alphaupdate(double *alpha,
                 double *resids,
                 double c,
                 int N,
                 double sigma2){
   int n;
   double meana, vara;
   meana=0.0;
   /* remove effect of alphaCur from the residuals*/
   for (n=0; n<N; n++ ){
      resids[n] += *alpha;
      meana += resids[n];
   }
   vara = c * sigma2/(N*c+ sigma2 );
   meana = vara * meana / sigma2;
   /* update alpha */
   *alpha = meana;
   /* add effect of alpha to residuals */
   for (n=0; n<N; n++ )
      resids[n] -= *alpha;
}
```
